# The Evolving Concepts of Haemodynamic Support: From Pulmonary Artery Catheter to Echocardiography and Theragnostics

**DOI:** 10.2174/157340311798220458

**Published:** 2011-08

**Authors:** Antonio Figueiredo, Nuno Germano, Pedro Guedes, Paulo Marcelino

**Affiliations:** CEDOC, Faculty of Medical Sciences, Lisbon

**Keywords:** Echocardiography, Intensive Care, teaching.

## Abstract

Echocardiography is a non-invasive tool, aimed towards the anatomical and functional characterization of the heart. In Intensive Care it is considered nowadays as a necessary tool for patient evaluation. However, the information obtained using echocardiography is not the same as provided by other means, namely the invasive ones. In recent years there has been a significant evolution in the general concepts of haemodynamic support for the critically ill patient. In this new environment, echocardiography has gained particular relevance. In this text the new positioning of echocardiography in the light of the new concepts for hemodynamic support is described, as well as, the need for a specific formative program directed towards Intensive Care physicians. A new generation of biomarkers can also add relevant information and start a new era in haemodynamic support. They may help to further characterize the disease process, identifying patients at risk, as well as, characterize specific organ failure as well as monitoring therapy.

## INTRODUCTION

The most serious challenge that faces clinical medicine is changing the natural course of disease avoiding, if possible, it’s most undesirable effect: death. In Intensive Care, a relatively new medical discipline, patients are in critical clinical conditions, with their biological systems on the physiological limits, in a life-threatening condition. Advanced life support is necessary to overcome situations, which, desirably, are reversible. 

Haemodynamic evaluation is a fundamental part of clinical practice. Major advances were made in the 1970’s with the introduction in the clinical practice of the invasive monitoring with pulmonary artery catheter (PAC). The concept was invented by Forssman in 1929 [1], and was later developed in England [2]. The floating catheter appears in 1953 [3], and soon afterwards was described a catheter similar to the one currently used [4]. Swan, Ganz and co-workers [5], showed that it was possible to perform this procedure at the bedside, obtaining several haemodynamic variables. This fact was important because at that time this device was considered dangerous, and confined to very specific areas inside hospitals, mainly for the diagnosis of congenital cardiopathies. In 1976, It was approved as a technical device not essential for life without the need for validation studies.

Much of the pathophysiology of critical illness that we know today was established with the help of this device. The information provided is based on different assumptions: intracardiac pressures that, in practice, represent the intravascular volume status; cardiac output obtained using the thermodilution technique; a series of related parameters, such as vascular resistances; oxygen transport variables considered important to provide organs and peripheral tissues of nutrients which stand as a measure of adequacy of nutrient supply to organs (Table **1**). 

We will consider these concepts and associated clinical practice as the first step of modern concepts in haemodynamics. They can be also considered as parameters of central haemodynamics or macro haemodynamics since they are obtained centrally within cardiac cavities, reflecting the physiological characteristics of this location, and are nonspecific for any particular organ.

Obtaining supraphysiological values of the variables has become the rule, turning the device PAC a treatment guide more than a diagnostic tool. The presuppositions for these attitudes were based on the initial observation that in critically ill patients a hyperdynamic pattern was typical of the survivors of acute illness [6-10]. Through a set of medical actions, the Intensivist tried to modify the haemodynamic profile of patients in an attempt to increase their survival. Moreover, it was thought that the acute nutrient requirements were substantially greater, facing the increased need of a stressed subject. A standard practice was established in order to obtain values above normal of intracardiac pressures and oxygen transport variables. For this purpose, therapeutic endpoints were generally similar: first fluids were administered (crystalloids or colloids) until the pulmonary artery wedge pressure reached 15 mmHg or 18 mmHg; if this intervention was not enough to increase the oxygen supply in more than 600 ml/min/m^2^, dobutamine was started to obtain a cardiac output greater than 4 or 4.5 litres per minute. As a last intervention, packed red blood cells were transfused in order to reach the desired value of oxygen transport. The volume of administered fluids reached high levels, sometimes 10 litres / day, and understandably, the number of interventions and medical prescriptions per patient were high [11].

We can say that monitoring using the pulmonary artery catheter became one of the technological hallmarks of Intensive Care. It can be comparable to endoscopes for gastroenterology, and so on. This symbolic-cultural aspect should not be bleached; presently, it has a major impact on the positioning and visibility of different medical specialties. This discussion is, however, beyond the scope of this text. Monitoring of patients has become "heavy", the amount of medical interventions were high, as there was a natural trend to normalize each of the physiological variables continuously monitored. Critical care medicine overplayed monitored parameters. Patients’ evaluation at bedside was clearly underestimated. There was an attempt to deal with objective tools, excluding the subjectivity of the observer.

## CRITIQUES TO THE MODEL

In cardiology, it quickly became apparent that treating patients in post acute myocardial infarction using the pulmonary artery catheter led to increased mortality [12-17]. 

In September 1996, 20 years after the introduction of the pulmonary artery catheter in clinical practice, Connors *et al* [18] published a randomized controlled study that urged the scientific community to reconsider the issue of invasive monitoring with PAC.

The finding or suggestion that routine use of PAC could induce higher mortality was a “concussion in the midst of Intensive Care”. These data were reviewed, and methodological robustness of the study by Connors *et al*, successively revised by experts, was never questioned. In contrast, data were successively confirmed in subsequent years by further studies conducted according to higher methodological standards [19-23].

The use of oxygen transport variables was also questioned, in multicenter, randomized studies, which may be regarded as a reference. Hayes *et al* [24], studied 100 patients in two Intensive Care units, 50 in a control group and 50 in a therapy group aimed to achieve supraphysiological values of cardiac output, oxygen transport and consumption. They found a significant increase in mortality within the therapeutic group (34% vs. 54% p = 0.04). But the most emblematic study was performed by Gattinonni *et al* [25], who studied 252 patients in a control group (goal: obtain a cardiac output between 2.5 and 3.5 litres / min), 253 patients in an intervention group (to achieve a cardiac index> 4.5 litres/m2) and 257 patients in another intervention group (to achieve a mixed venous saturation> 70%), enrolled in 56 Intensive Care units. There were no statistically significant differences in mortality between groups; although patients in the second group had higher mortality (48%, 52% and 48% respectively); other studies have confirmed these findings [26-28]. 

Using appropriate methodologies, Sandham *et al* [29], performed a study much like the initial studies of Shoemaker on optimizing preoperative haemodynamic parameters in patients undergoing major surgery, and sustained in large measure the initial concepts, briefly addressed. They studied 997 patients in each arm and with sound methodology. Their findings are opposite to those of Shoemaker, highlighting that methodological issues are not negligible, especially if the aim of a clinical trial is to change clinical practice. Transfusion support also suffered major criticism [30]. Nowadays, the most appropriate levels of haemoglobin in critical patients (without coronary heart disease) are between 7 and 9 g / dL. These data hardly justify an aggressive transfusion policy and are in clear contradiction with previous practices.

The practices of fluid administration changed considerably. The notion that the administration of fluids may be beneficial in critical situations such as, septic shock cannot be considered universally accepted [31-34]. As dynamic concepts of fluid responsiveness were accepted, the fluid restriction is recommended and the deleterious effects of fluid overload are recognized [35-40].

Presently it is consensually agreed that the use of a pulmonary artery catheter in critically ill patients is irrelevant to the prime indicators of outcome, morbidity and mortality. Another message arose: in order to change an established practice, the scientific evidence must meet high-quality requirements. 

## THE NEW PARADIGMS

In the scientific community the need for new paradigms of haemodynamic support was felt. Precisely 10 years after the publication of Connors *et al*, in September 2006, the consensus conference on haemodynamic monitoring of the European Society of Care Medicine held in Paris [41]. The resulting document, published in 2007, reflects the maturation process of  new ideas and concepts, which eventually changed and shaped the modern perspectives on the haemodynamic approach of critically ill patients.

The main conceptual changes can be briefly stated. Recalling the assumptions stated for the pulmonary artery catheter, the critiques can be made either to the parameters based on pressure, recognizing their relative value as indicators of intravascular volume, and critiques to parameters based on oxygen transport variables.

The intravascular volume should be tailored to each patient and to each stage of the disease. The intracardiac pressures are helpless in this regard. The concept of dynamic parameters of response to the fluid, which echocardiography contributed to, indicates that fluids administered should remain in the intravascular space (this is what really matters, because the swelling of organs is deleterious and contributes to organ dysfunction). That is, the fact that a patient has a central venous pressure of 2 mmHg or 10 mmHg, or equivalent wedge pressure, is not indicative that the administration of fluids is beneficial or not. The current concepts establish that in an individual patient, an effective volume that meets the needs at a specific disease stand point is desirable. The adverse effects of either deficit or volume excess should be avoided.

Cardiac output remained a parameter of some value in Intensive Care. It is essential for determining the haemodynamic profile in hypotension and subsequent treatment, and can also assist in the administration of fluids: a quantity of fluid remains in the intravascular space, if it would result in an increase in cardiac output. Another question pertains to the real need for a continuous monitoring of cardiac output in the critically ill, and if it is superior to intermittent measurements.

A return to clinical observation parameters seems also obvious. The adequacy of organ perfusion is based on serum lactate levels, urine output and level of consciousness. Hence, patient evaluation was driven from the monitor to the patient himself. 

It is assumed that initially there was a willingness to address the patient based on mechanistic concepts of whole-body function, which has progressively evolved towards more individualized, relativistic concepts.

We consider these concepts as the second stage of the recent evolution of modern haemodynamic concepts. Nonetheless, affirm that echocardiography is a technique specially adapted to this new thinking, not dependent on intracardiac pressures, more clinically comprehensive because it does not restrict the patient to a set of critical variables on a monitor.

Echocardiography has always been coveted for Intensive Care [42,43]. Its journey and the role that it has been given within the specialty meet the thought of each era. Three patterns of use can be distinguished, in accordance to the concepts at their time of appearance. In the beginning there was a particular concern to reproduce the intracardiac pressures with the use of echocardiography, using echocardiography as a non-invasive PAC. The evolution of the relative positioning of echocardiography within Intensive Care is demonstrated in Table **2**. However, several problems were identified at this time [44]. Tables **3** and **4**  summarize a number of equations and means to determine pulmonary capillary wedge pressure [45-63]. The amount and complexity of these demonstrate that this path can hardly be accepted as a routine in critically ill patients. Most of them were obtained outside the Intensive Care Unit (ICU), in non-critical conditions, in studies conducted in echocardiography laboratories. These conditions and patients are hard to reproduce in the ICU, where most patients are non-cooperative and under mechanical ventilation. In a recent publication, in 704 ICU patients, the possibility of detecting E/A mitral wave for analysis was 85.9% of patents in sinus rhythm [64]. Moreover in that cohort 23% of patients were in atrial fibrillation and 19% presented a heart rate over 120 beats per minute. Thus, the condition to perform an adequate analysis of Doppler-derived parameters is limited at the bedside.

To echocardiography was further reserved the role of examination, by request of the Intensive Care physician in case of suspected heart condition. These examinations on demand could not have a real impact on daily practice. This is not a real-time performance and did not answer the usual problems of critical situations. Finally came up studies directed to critically ill patients, performed by Intensive Care specialists, studying patients at the bedside, with the primary intention of responding to specific issues: the pathophysiology of septic shock, the study of diastolic dysfunction in sepsis, and so on [65,67].

Since the beginning it was known that the strategy based on the approach to the patient by echocardiography wouldn’t depend on intracardiac pressures. It implies a different approach to the patient. In the era of invasive monitoring, certainly a degree of disbelief was noticed. But the recent changes in haemodynamic concepts, briefly discussed above, turned the acceptance of echocardiography easier and the margin of progression of echocardiography in Intensive Care medicine higher.

In regard to the problems chronically pointed out in echocardiography, the necessary learning curve is perhaps the most frequently reported. It will be necessary to provide training that permits access to the technique. Currently, stepwise training is accepted and even accredited in several countries, from the simple fast examinations to more complex examinations performed by skilled Intensive Care physicians [68].

Furthermore, echocardiography could not add any prognostic value to the usual scoring systems used in medicine. Rough haemodynamic variables demonstrated its value in cardiogenic shock [69] and Sturgess *et al* [70] observed that tissue-Doppler derived parameters were independently associated with mortality, along with fluid balance and APACHE III scoring system. This study, however, enrolled only 21 patients with sepsis in an ICU. Other studies were also inconclusive, so there is no evidence-based information to support the prognostic relevance of echocardiography.

## BIOMARKERS AND CRITICAL ILLNESS: HOW HELPFUL ARE THEY?

Some questions cannot be addressed using the usual tools for haemodynamic support. End organ failure and multi-organ failure are common features in the ICU. They are linked to poor outcomes and present a challenge to the physician, both in anticipating events, and providing adequate treatment. The aetiology is not as uniform as the reaction of the organism to an insult. Indeed, this reaction seems to be a stereotype in the responsiveness to aggression. A high number of acute phase molecules could be described, but they are not disease specific or organ specific [71]. They are linked to pathways, common in many disease processes, like non-specific inflammatory response and apoptotic phenomena.

A number of characterization and prognostic scoring systems had been described. The well known APACHE, SAPS, SOFA and MODS [72-75] are based on simple organ failure measurements and are widely used. The notion of organ failure is therefore critical in the Intensive Care. But the known describing systems are not quite accurate. An effort on the part of the investigators towards the description of (a) biomarker(s) for the situations is quite documented. The monitoring devices can characterize serial variables concerning heart pressures, cardiac output and oxygen transport and consumption. But the concepts that relied on a mechanistic understanding of human functioning in critical illness are probably outdated, although still playing a significant role in common educational programs in the field. 

The growing number of inflammatory markers described did not answer the main question for organ specific or even disease specific markers. Available data make them unreliable to replace old haemodynamic parameters as guides for major therapeutic actions, such as fluid or vasopressor prescription. And they could help in defining the adequacy of circulating volume or ongoing therapeutic actions, regardless of blood pressure, central venous or cardiac output values.

## BIOMARKERS OF CARDIAC LESION IN INTENSIVE CARE

Biomarkers of myocardial lesion were found to be useful in Intensive Care. The most extensively studied biomarker is troponin I, which has a consolidated place in the diagnostic of myocardial necrosis. Babuin *et al* [76] studied 1637 critically ill patients, with a mortality of 12.5%. They divided all patients into those with serum troponin I < 0.01 and those with troponin I > 0.01. The authors described a significantly higher mortality in patients with troponin I > 0.01, during ICU stay, at 30 days and even at 3 years. The same observations were described by Stein *et al.* [77], who verified that even small increases in troponin I (between 0.1 and 1.49) were related to higher mortality in the ICU (5% vs. 28%). These authors did not find a difference in ICU stay or mortality at 6 months. In another setting, Vasile *et al* [78] studied 1076 patients with upper gastrointestinal haemorrhage and verified that higher troponin I levels were related to higher mortality. The difference in mortality maintained at 30 days and 3 years. 

In patients with sepsis there is considerable evidence that troponin I levels are related to poorer outcome in the ICU. In Ttable **5** a sum of 8 studies conducted in these patients is presented [79-86]. There is evidence suggesting a relationship between troponin I levels and mortality in critical patients. It`s relevant that this association can identify patients with cardiac dysfunction due to sepsis. One study revealed a lack of correlation between troponin I levels and cardiac output. Although only 10 patients were enrolled, we found this study interesting due to the following questions regarding sepsis-related cardiomyopathy: the usual haemodynamic pattern of sepsis patients with cardiac dysfunction is not altered (normal or high cardiac output and low peripheral vascular resistance); so only a few methods can identify these patients (the serum troponin I levels and probably echocardiography). It means that if none of these examinations is performed, the situation may go undetected.

Other markers of cardiac disease were also tested. One of them is N-terminal pro BNP levels. Rudiger *et al* [87] compared 2 groups: patients with septic shock and other patients with heart failure without sepsis. The NT-BNP levels increased in both groups, although they presented quite distinctive haemodynamic profiles. Charpentier *et al*. studied a small cohort of sepsis patients (n=9), and verified that in deceased patients there existed a trend for higher pro-BNP levels. In Table **6** studies that relate sepsis and pro-BNP levels are presented [88-93].

The relation between pro-BNP levels and outcomes in the critically ill were not strong enough, a clear relation with mortality could not be found. However, Sturgess *et al* [70] observed that tissue-Doppler E/e´ had prognostic value in sepsis patients. They also demonstrated that this parameter was also related to pro-BNP levels. The relative importance of diastolic dysfunction incritical illness is thus suspected, but not proved. The aetiology of this kind of disturb is not fully understood, nor is the role of specific therapy aimed to counteract with it.

It should be stressed that  myocardial injury in sepsis is not related with myocardial necrosis or coronary disease. In fact, only a modest increase in troponin I, barely seen in myocardial infarction, is enough to distinguish patients at higher risk.

The presented data can reach high significance for risk stratification and possible management of critically ill patients. They also demonstrate that some biochemical markers of organ damage may be useful in the overall assessment of critical care patients. As evidence, information provided by these markers, in particular troponin I, is not related to the usual haemodynamic information obtained with several monitoring tools, such as, cardiac output or peripheral vascular resistance. The information provided by these markers can actually reflect the end organ damage, mediated by the cascade of inflammatory events, typical of sepsis and other pathological conditions of the critically ill. Although its use can be considered, these markers did not become part of a routine evaluation of the critically ill. 

## THE MULTIORGAN FAILURE AND BIOMARKERS

In the case of cardiac lesion in the critical care setting, troponin I can be a good marker of end organ damage and is now extensively studied. But the critically ill often present multiple organ failure, involving the lungs, liver, central nervous system, bowel, kidneys, and so on. The usual and recognised markers of organ damage (serum lactate, mixed venous saturation, and central haemodynamic parameters, e.g., cardiac output and peripheral vascular resistance) are not organ specific and appear late in the course of the disease. In the case of  kidney injury, new markers of early damage may be useful and will be advantageous over the usual markers (serum creatinine, BUN or urine output) [94, 95], but for other organs there are no established lesion markers. Citrulline may be a useful marker of enteric function, a critical organ in the Intensive Care [96, 97], but exclusively used as a diagnostic biomarker. The plasma levels of citrulline are related to the global enteric mass. In the critically ill, Piton *et al.* [98] performed a study in 67 patients without bowel disease and without renal disease, observing that low levels of citrulline at 24 hours were related to high plasma C-reactive protein, nosocomial infection, and 28-day mortality. There is no other comparable marker of bowel failure in use. The best possible approach to it is stasis and the impossibility to feed using feeding devices. 

The distinction between systemic inflammation response syndrome (SIRS) and infection related inflammation (sepsis) is one of the most striking features in Intensive Care. The haemodynamic profile is similar in both situations, mainly characterized by a decrease in systemic vascular resistance and increased cardiac output. Complex measurements can be performed, like the description of the matrix metalloproteases and their inhibitors [99]. Leptin was also described to distinguish SIRS from sepsis, matching the levels of tumour necrosis factor-alpha [100]. Currently, one can perhaps consider C-reactive protein (CRP) and procalcitonin (PCT) the most popular biomarkers of infection. Generally, PCT is considered superior to CRP for distinguishing SIRS form sepsis [101-103]. In the recent published PRORATA study [104], conducted in 630 patients, 319 in control group and 311 in procalcitonin group demonstrated that patients in the PCT group had more antibiotic-free days, thus guiding treatment. However, despite the growing data supporting the use of this biomarker, both as diagnostic parameters, and as guiding therapy parameters, some investigators could not find a clear superiority over the basic clinical parameters [105]. Regarding a sensitive matter like fluid therapy, it is now generally accepted that excess fluid can be harmful and contribute to end-organ failure [106-109]. The methods in use to guide fluid therapy lack sensitivity, and the new dynamic concepts could not be applied to a large number of patients. To date, no investigation was performed in order to test the usefulness of a biomarker in guiding fluid therapy. 

This ability of biomarkers to guide therapy is perhaps one of the most interesting features. The description of biomarkers as diagnosetic and therapeutic tools was gained a definite place in our understanding of critical illness. An important study by Wenkui *et al.* [110] considered the issue of the well recognized beneficial effect of fluid restriction. In 299 patients undergoing gastrointestinal surgery they adjusted the fluid therapy by serum lactate levels as a marker of global organ perfusion. They observed that patients with a restrictive fluid regimen adjusted by serum lactate presented a lower rate of complications, but the patients who needed more fluids presented the highest rate of postoperative complications. A diagnostic test that allows the identification of patients at risk, that helps target therapy, along with the monitoring of the response, is a desirable tool for our future. This global approach is known as theagnostics [111-114], and probably represents the next stage of haemodynamic support. Genomics, proteomics, and other recent disciplines are the major contributors. In the future they probably will shape our concepts on disease process, from diagnosis to therapeutics and monitoring. The authors present theragnostics as a form of detecting patients at risk, especially through genomic analysis.  Perhaps the use of these novel biomarkers is more than that. They could help the physician to more accurately characterize the disease process at bedside, much beyond the central haemodynamic parameters (central venous pressure, pulmonary capillary wedge pressure, cardiac output, oxygen delivery, mixed venous saturation, left ventricular performance), and also characterize the specific organ damage, using organ-specific biomarkers. Note that the assessment of organ damage is performed mainly using serum lactate measurements and its kinetics, urine output and consciousness level. A regular measurement could provide the desirable monitoring ability. In Table **7** the information provided by each approach is summarized. Note that to date there is no single method capable of giving all needed information. Even with the advent of new parameters based on molecular medicine come “old” tests (like echocardiography) should prevail. 

Nonetheless, the recent lessons from the PAC must not be forgotten: close patient evaluation is always critical over new technology. Moreover, the technological approaches must demonstrate their superiority over the old ones. These processes are followed in the pharmaceutical industry and if PAC was a medication, perhaps it would have never entered the market. The number of physiological variables monitored is related to the number of medical interventions performed [115]; and the number of medical interventions may be linked to iatrogenic burden.

Another sensitive question pertains to the feasibility with regards to the set of biomarkers to use. This practical question should not be overlooked. All tests must be worthy in different domains: scientific, economical and technical. There are no available studies on this matter, as the molecules described were used in an investigational context, not in a massive fashion.

The way we can use these markers is not yet established. How can they influence patient management or outcome? Can we use them as guiding parameters both for diagnostic and therapeutic measures? Theoretically they may be able to individualize the therapeutic actions, apart from the normalization of rude haemodynamic parameters, representing a rather mechanistical approach to the patients. Much work remains to be done in order to go further in the haemodynamic concepts of patient management, towards a better care of the critically ill.

## CONCLUSION

It’s absolutely breathtaking to appreciate the scope of future progress in haemodynamic concepts that allow us to visualize a third level in a not too distant future. Scientific research, with the multiple possibilities that are offered today, will contribute substantially to these advances. Currently recommended strategy to assess the adequacy of circulating volume and the effective volume is still very poor. If, as we know, there are haemodynamic parameters that help us in its determination, the evaluation is limited to the recommended levels of serum lactate, urine output, and consciousness. New biomarkers related to organ perfusion and function can add important elements in the future, guiding both diagnosis and treatment.

## Figures and Tables

**Table 1.** Reference Values for Main Haemodynamic Variables Obtained Using PAC

Parameters	Limits	Units
CVP	1-6	mmHg
PCwP	6-12	mmHg
CI	2.4-4.0	l/min/m^2^
LVWI	40-60	g.m/m^2^
RVWI	4-8	g.m/m^2^
SVRI	1600-2400	dyn.sec.m^2^/cm^5^
PVRI	200-400	dyn.sec.m^2^/cm^5^
S_V_O_2_	70-75	%
TO_2_	520-570	ml/min/m^2^
VO_2_	110-160	ml/min/m^2^
EO_2_	20-30	%

**Legend:** CVP, central venous pressure; PCwP, pulmonary capillary wedge pressure; CI, cardiac index; LVWI, left ventricular work index; RVWI, right ventricular work index; SVRI, systemic vascular resistance index; PVRI, pulmonary vascular resistance index; SvO_2_, mixed venous oxygen saturation; DO_2_, oxygen delivery; VO_2_, oxygen consumption; EO_2_, oxygen extraction.

**Table 2.** Comparison of the Evolution of Haemodynamic Concepts and the Relative Positioning of Echocardiography in Intensive Care

Time	Main Diagnostic Tool	Role of Echocardiography	Echo-equipments
Until 90s	Pulmonary artery catheter	No more than a curiosity	Heavy, low portability
Mid-90s	Pulmonary artery catheter	Attempt to use it as a non-invasive PAC Performance on-demand to exclude specific diagnosis	Easier to transport
Early XXI century	Several devices (mixed venous saturations, continuous cardiac output, echocardiography...)	ICU physicians perform echocardiograms according to their specific needs Need for specific formative programs in this field	Highly portable equipments High performance equipments

**Table 3.** Equations for Calculation of Pulmonary Capillary Artery Wedge Pressure Using Echocardiography

Authors	n	Main Disease	Equation
Vanovershelde [45]	132	Cardiac	18,4+17,1(inverse mitral E/A)
Chirillo [46]	58	Cardiac	94,261(tdFP-9,831) -16,337durFP+44,261
Gozalez-Vilchez [47]	54	Heart surgery	(1000/2xTRIV+FPV) x4,5-9
Temporelli [48]	35	Cardiac	51-0,26(E/Am)
Garcia [49]	45	Cardiac and sepsis (n=7)	5,2x (velpEm/FPV) +4,6
Nagueh [50]	125	Cardiac	1,24x (velpEm/Ea)+1,9
Nagueh [51]	49	Cardiac	17+(5,3E/Am) -0,11 (TRIV)
Mulvagh [54]	41	Cardiac	46-0,22TRIV-0,1dAm-0,003tdEm
Nagueh [53]	42	Cardiac	22+0,005velpEm-0,183TRIV
Cláudio David [54]	¿n??	Cardiac	(1000xTAc/TEj) - (dur a-A)

Legend: E/Am, mitral E/A; DTpv, pulmonary vein deceleration time; durFP, length of pulmonary vein flux; TRIV, left ventricular isovolumetric relaxation time; FPV, mitral flow propagation velocity; velpEm, mitral E wave pick velocity; tdEm, mitral E wave deceleration time; TAc, pulmonary artery acceleration time; TEj, pulmonary artery ejection time; dur a, right superior pulmonary vein systolic wave time; A, mitral A wave time. Cardiac diseases include patients with coronary artery disease, dilated cardiomyopathy and valvular diseases.

**Table 4.** Studies Establishing Different Correlations Between Echocardiographic Parameters and Pulmonary Capillary Wedge Pressure, Not Resulting in Equations.

Authors	n	Patient Characteristics	Conclusion
Tenenbaum [55]	55	Dilated cardiomyopathy and a control of normal volunteers	Inverse correlation between mitral A wave deceleration time and PCwP (p< 0.0001)
Nishimura [56]	97	Patients with systolic dysfunction (group A) and hypertrophic cardiomyopathy(group B)	Data from patients with systolic dysfunction could not be applied to patients with hypertrophic cardiomyopathy
Nagueh [57]	45	Patients with hypertrophic cardiomyopathy	PCwP was related to A/Ar relation, and E/Ea relation
Chezbraum [58]	53	Patients with cardiac diseases and a control group	IVRT (inverse relation, p< 0.001); if E/A>1, then PCwP>15
Appleton [59]	76	Patients with coronary artery disease	A dilated LA was related to high PCwP. A high PCwP was related to increased mitral E/A, to DTEm (inverse relation) and IVRT (inverse relation)
Pozzoli [60]	49	Dilated cardiomyopathy awaiting cardiac transplantation	A high PCwP was related to mitral E/A and IVRT (inverse relation)
Brunazzi [61]	96	Coronary artery disease and valvular heart disease patients	A qualitative assessment of PCwP is possible through analysis of pulmonary veins in a transthoracic approach
Masayama [62]	28	Coronary heart disease, valvular heart disease and dilated cardiomyopathy patients	DTEm modified with the treatment of CHF
Giannuzzi [63]	152	Patients with acute coronary syndrome	If mitral E/A>2, then PCwP>20mmHg. If DTEm<120ms, then PCwP> 20mmHg

Legend. PCwP- pulmonary capillary wedge pressure; LA, left atrium; IVRT, left ventricular isovolumetric relaxation time; DTEm- mitral E wave deceleration time; CHF, congestive heart failure

**Table 5.** Data from 8 Clinical Studies Aimed to Relate Troponin I Levels and Outcome in Sepsic Patients

Patients (n)	239
Sepsis patients (n)	207
Association between troponin I levels and mortality (n)	207
Association between troponin I levels and myocardial dysfunction (n)	117 (4 studies)
Lack of relation between troponin I levels and cardiac output (n)	10 (1 study)

**Table 6.** Data from 6 Clinical Studies Relating to Pro-BNP Levels and Sepsis

Patients (n)	253
Sepsis patients (n)	170
Association with left ventricular dysfunction	21 (2 studies)
Association with mortality	51 (2 studies)

**Table 7.** Information Provided by Known Parameters and Devices and the Future Information Possibly Provided by New Generation Biomarkers and Theragnostics.

	Pulmonary Artery Catheter and Continuous Cardiac Output	Mixed Venous Saturation and Serum Lactate	Echocardiography	Inflammation and Aptoptosis Biomarkers	Theragnostics
Establishing haemodynamic profile	+	-	+	-	-
Identification of patients at risk	-	-	-	+	+
Multi-organ failure	+/-	+/-	+/-	+	+
Specific organ failure	-	-	-	-	+
Guiding therapy	+/-	+/-	+/-	-	?

Legend: +, yes; +/-, not evident; -, no.

## References

[1] Forssman W (1929). Die Sondierung der rechten hertzens. Berlin Klin Wochenschr.

[2] Branthwaite MA, Bradley RD (1948). Measurement of cardiac output by thermal dilution in man. J Appl Physiol.

[3] Lategola M, Rahn H (1953). A self-guided catheter for cardiac and pulmonary arterial catheterization and occlusion. Proc Soc Exp Biol Med.

[4] Bradley RD (1964). Diagnostic right-heart catheterization with miniature catheters in severely ill patients. Lancet.

[5] Swan HCJ, Ganz W, Forrester Marcus H, Diamond G, Chonette D (1970). Catheterization of the right heart in man with use of a flow-directed balloon-typed catheter. N Eng J Med.

[6] Shoemaker WC (1972). Cardiorespiratory patterns of surviving and non-surviving postoperative surgical patients. Surg Gynecol Obstet.

[7] Shoemaker WC, Montgomery ES, Kaplan E (1973). Physiologic patterns in surviving and non-surviving shock patients: use of a sequential cardiorespiratory parameters in defining criteria for therapeutic goals and early warning of death. Arch Surg.

[8] Shoemaker WC, Appel PL, Bland R (1983). Use of physiologic monitoring to predict outcome and to assist in clinical decisions in critically ill post operative patients. Am J Surg.

[9] Shoemaker WC, Appel PL, Kram HB, Waxman K, Lee T-S (1988). Prospective trial of supranormal values of survivors as therapeutic goals in high-risk surgical patients. Chest.

[10] Tuchschmidt J, Fried J, Astiz M, Rackow E (1992). Elevation of cardiac output and oxygen delivery improves outcome in septic shock. Chest.

[11] Waxman A, Ward N, Thompson T (2005). Round?table debate: Controversies in the management of the septic patient - desperately seeking consensus. ccforum.com/content/9/1/E.

[12] Connors AF, MacAffree DR, Gray BA (1983). Evaluation of right-heart catheterization in the critically ill patient without acute myocardial infarction. N Eng J Med.

[13] Hocham JS, Boland J, Sleeper LA (1995). Current spectrum of cardiogenic shock and effect of early revascularization on mortality. Circulation.

[14] Costa JTS, Laureano Santos A, Melo M (1992). Influence of the use of Swan-Ganz catheter on mortality from acute myocardial infarction. Rev Port Cardiol.

[15] Zion MM, Balkin J, Rosenmann D (1990). Use of pulmonary artery catheters in patients with acute myocardial infarction. Analysis in 5841 patients in the SPRINT registry. Chest.

[16] Gore JM, Spodick DH, Goldberg RJ, Alpert J, Dalen D (1987). A community-wide assessment of the use of pulmonary artery catheters in patients with acute myocardial infarction. Chest.

[17] Yu M, Takanishi D, Myers SA (1995). Frequency of mortality and myocardial infarction during maximizing oxygen delivery. Crit Care Med.

[18] Connors AF, Speroff T, Dawson N (1996). The effectiveness of right heart catheterization in the initial care of critically ill patients. JAMA.

[19] Polonen P, Ruokonen E, Hippelainen M, Poyhonen M, Takala J (2000). A prospective, randomized study of goal-oriented hemodynamic therapy in cardiac surgical patients. Anaesthesia & Analgesia.

[20] Gattinoni L, Brazzi L, Pelosi P (1995). A trial of goal-oriented hemodynamic therapy in critically ill patients. N Eng J Med.

[21] (2005). ESCAPE investigators Evaluation study of congestive heart failure and pulmonary artery catheterization effectiveness. JAMA.

[22] Rhodes A, Cusak RJ, Newman PJ, Grounds M, Bennett ED (2002). A randomised, controlled trial of the pulmonary artery catheter in critically ill patients. Intensive Care Med.

[23] Shah MR, Hasselbad V, Stevenson LW (2005). Impact of the pulmonary artery catheter in critically ill patients. JAMA.

[24] Hayes MA, Timmins AC, Yau EH, Polazzo M, Watson D, and Hinds CJ (1997). Oxygen transport patterns in patients with sepsis syndrome or septic shock. Crit Care Med.

[25] Gattinoni L, Brazzi L, Pelosi P (1995). A trial of goal-oriented hemodynamic therapy in critically ill patients. N Eng J Med.

[26] Durham R, Neunaber K, Mazuski JE, Shapiro M, Baue A (1996). The use of oxygen consumption and delivery as end-points for resuscitation in critically ill patients. J Trauma.

[27] Hayes MA, Timmins AC, Yau EH, Palazzo M, Hinds C, Watson D (1994). Elevation of systemic oxygen delivery in the treatment of critically ill patients. N Eng J Med.

[28] Heyland DK, Cook DJ, King D (1996). Maximizing oxygen delivery in critically ill patients: a methodological appraisal of the evidence. Crit Care Med.

[29] Sandham JD, Hull RD, Brant RF (2000). A randomized controlled trial of pulmonary artery catheter use in 1994 high-risk geriatric surgical patients. Crit Care Med.

[30] Hébert PC, Wells G, Blajchman MA (1999). A multicenter, randomized, controlled clinical trial of transfusion requirements in critical care. N Eng J Med.

[31] Durairaj L, Schmidt G (2008). Fluid therapy in resuscitated sepsis. Less is more. Chest.

[32] Payen D, de Pont A, Sakr Y (2008). A positive fluid balance is associated with a worse outcome in patients with acute renal failure. Crit Care.

[33] Alsous F, Khamiees M, DeGirolamo A, Amoateng-Adjepong Y, Manthous CA (2000). Negative fluid balance predicts survival in patients with septic shock: a retrospective pilot study. Chest.

[34] Varon J, Fromm RE (2000). Fluid balance in sepsis.Are we ready for a negative balance?. Chest.

[35] Teboul J-L (2003). Dynamic concepts of volume responsiveness. Intensive Care Med.

[36] Tavernier B, Makhotine O, Lebuffe G, Dupont J, Scherpreel P (1998). Systolic pressure variation as a guide to fluid therapy in patients with sepsis-induced hypotension. Anesthesiology.

[37] Michard F, Boussat S, Chemla D (2000). Relation between respiratory changes in arterial pulse pressure and fluid responsiveness in septic patients with acute circulatory failure. Am J Respir Crit Care Med.

[38] Marx G, Cope T, McCrossan L (2004). Assessing fluid responsiveness by stroke volume variation in mechanically ventilated patients with severe sepsis. Eur J Anaesthesiol.

[39] Barbier C, Loubieres Y, Schmit C (2004). Respiratory changes in inferior vena cava diameter are helpful in predicting fluid responsiveness in ventilated patients. Intensive Care Med.

[40] Feissel M, Michard F, Mangin I, Ruyer O, Faller J-P, Teboul JL (2001). Respiratory changes in aortic blood flow velocity as an indicator of fluid responsiveness in ventilated patients with septic shock. Chest.

[41] Antonelli M, Levy M, Andrews PJ (2006). Hemodynamic monitoring in shock and implications for management. Intensive Care Med.

[42] Cholley B, Vieillard-Baron A, Mebazaa A (2005). Echocardiography in the ICU: time for widespread use. Intensive Care Med.

[43] Beaulieu Y, Marik P (2005). Bedside ultrasonography in the ICU. Part 1. Chest.

[44] Marcelino P, Lopes MG (2006). Non invasive determination of pulmonary wedge pressure using echocardiography. Acta Medica Port.

[45] Vanovershelde J-L, Robert AR, Gerbaux A (1995). Noninvasive estimation of pulmonary arterial wedge pressure with Doppler transmitral flow velocity pattern in patients with known heart disease. Am J Cardiol.

[46] Chirillo F, Brunazzi MC, Barbiero M (1997). Estimating mean pulmonary wedge pressure in patients with chronic atrial fibrillation frm transthoracic Doppler indexes of mitral and pulmonary venous flow velocity. J Am Col Cardiol.

[47] Gonzalez-Vilchez F, Ares M, Ayuela J (1999). Combined use of pulsed and color M-mode Doppler echocardiography for the estimation of pulmonary capillary wedge pressure: an empirical approach based on an analytical relation. J Am Coll Cardiol.

[48] Temporelli PL, Scapellato F, Corrá U (1999). Estimation of pulmonary wedge pressure by transmitral Doppler in patients with chronic heart failure and atrial fibrillation. Am J Cardiol.

[49] Garcia MJ, Ares MA, Asher C (1997). An index of early left ventricular filling that combined with pulsed Doppler peak E velocity may estimate capillary wedge pressure. J Am Coll Cardiol.

[50] Nagueh SF, Middleton KJ, Kopelen HN (1997). Doppler tissue imaging: a noninvasive technique for evaluation of left ventricular relaxation and estimation of pressures. J Am Coll Cardiol.

[51] Nagueh SF, Kopelen há Zoghbi WA (1995). Feasibility and accuracy of Doppler echocardiographic estimation of pulmonary artery occlusive pressure in the Intensive Care Unit. Am J Cadiol.

[52] Mulvagh S, Quinones MA, Kleiman NS (1992). Estimation of left ventricular end-diastolic pressure from Doppler transmitral flow velocity in cardiac patients independent of systolic performance. J Am Coll Cardiol.

[53] Nagueh SF, Kopelen HA, Quinones MA (1996). Assessment of left ventricular filling pressures by Doppler echocardiography in the presence of atrial fibrillation. Circulation.

[54] Claudio David Ana Almeida José Morais (2001). Left ventricular diastolic pressure evaluated through pulmonary transvalvular and pulmonary venous flow using echocardiography. Correlation with hemodynamic data. Rev Port Cardiol.

[55] Tenenbaum A, Motro M, Hod H (1996). Shortened Doppler-derived mitral A wave deceleration time: an important predictor of elevated left ventricular filling pressure. J Am Coll Cardiol.

[56] Nishimura RA, Appleton CP, Redfield MM (1996). Noninvasive Doppler echocardiographic evaluation of left ventricular filling pressures in patients with cardiomyopathies: a simultaneous Doppler echocardiographic and cardiac catheterization study. J Am Coll Cardiol.

[57] Nagueh SF, Lakkis NM, Middleton KJ (1999). Doppler estimation of left ventricular filling pressures in patients with hypertrophic cardiomyopathy. Circulation.

[58] Chenzbraum A, Keren A, Stern S (1992). Doppler echocardiographic patterns of left ventricular filling in patients early after acute myocardial infarction. Am J Cardiol.

[59] Appleton CP, Galloway JM, Gonzalez MS (1993). Estimation of left ventricular filling pressures using two-dimensional and Doppler echocardiography in adult patients with cardiac disease. J Am Col Cardiol.

[60] Pozzoli M, Capomolla S, Pinna G (1996). Doppler echocardiography reliably predicts pulmonary artery wedge pressure in patients with chronic heart failure with and without mitral regurgitation. J Am Coll Cardiol.

[61] Brunazzi MC, Chirillo F, Pasqualini M (1994). Estimation of left ventricular diastolic pressures from precordial pulsed-Doppler analysis of pulmonary venous and mitral flow. Am Heart J.

[62] Masuyama T, Lee J-M, Nagano R (1995). Doppler echocardiographic pulmonary venous flow-velocity pattern for assessment of the hemodynamic profile in acute congestive heart failure. Am Heart J.

[63] Giannuzzi P, Imparato A, Temporelli PL (1994). Doppler derived mitral deceleration time of early filling as a strong predictor of pulmonary capillary wedge pressure in postinfarction patients with left ventricular systolic patients. J Am Col Cardiol.

[64] Marcelino PA, Marum SM, Fernandes AP, Germano N, Lopes MG (2009). Routine transthoracic echocardiography in a general Intensive Care Unit: an 18 month survey in 704 patients. Eur J Intern Med.

[65] Jardin F, Valtier B, Beauchet A, Dubourg O (1994). Invasive monitoring combined with two-dimensional echocardiographic study in septic shock. Intensive Care Me.

[66] Jardin F, Dubourg O, Bourdarias J-P (1997). Echocardiographic pattern of acute cor pulmonale. Chest.

[67] Jardin F, Vieillard-Baron A (2005). Monitoring right-sided heart function. Curr Opin Crit Care.

[68] Price S, Via G, Sloth E, Guarracino F, Breitkreutz R, Catena E, Talmor D (2008). Echocardiography practice, training and accreditation in the Intensive Care: document for the World Interactive Network Focused on Critical Ultrasound (WINFOCUS). Cardiovasc Ultrasound.

[69] Torgensen C, Schmittinger CA, Wagner S (2009). Hemodynamic variables and mortality in cardiogenic shock: a retrospective study. Crit Care.

[70] Sturgess DJ, Marwick TH, Joyce C (2010). Prediction of hospital outcome in septic shock: a prospective comparison of tissue Doppler and cardiac biomarkers. Crit Care.

[71] Pierrakos C, Vincent JL (2010). Sepsis biomarkers: a review. Crit Care.

[72] Knaus W, Draper E, Wagner D, Zimmerman J (1985). APACHE II: a severity of disease classification system. Crit Care Med.

[73] Le Gall JR (1993). (almost two authors more) A new simplified acute physiology score (SAPS II) based on a European/North American multicenter study. JAMA.

[74] Vincent J-L (1996). (almost two authors more) The SOFA (sepsis-related organ failure assessment) score to describe organ dysfunction failure. Intensive Care Med.

[75] Marshall JC, Cook DJ, Christou NV (1995). Multiple organ dysfunction score: a reliable descriptor of a complex clinical outcome. Crit Care Med.

[76] Babuin L, Vasile VC, Peres JA (2008). Elevated cardiac troponin is an independent risk factor for short- and long-term mortality in medical Intensive Care unit patients. Crit Care Med.

[77] Stein R, Gupta B, Agarwal S (2008). Prognostic implications of normal
(<0.10ng/ml) and borderline (0.10 to 1.49 ng/ml) troponin elevation
levels in critically ill patients without acute coronary syndrome. Am J Cardiol.

[78] Vasile VC, Babuin L, Peres JA (2009). Long-term prognostic significance of elevated cardiac troponin levels in critically ill patients with acute gastrointestinal bleeding. Crit Care Med.

[79] Fernandes CJ Jr, Akamine N, Knobel E (1999). Cardiac troponin: a new serum biomarker of myocardial injury in sepsis. Intensive Care Med.

[80] Spies C, Haude V, Fitzner R (1998). Serum cardiac troponin T as a prognostic marker in early sepsis. Chest.

[81] Turner A, Tsamitros M, Bellomo R (1999). Myocardial cell injury in septic shock. Crit Care Med.

[82] Arlati S, Brenna S,  Prencipe L (2000). Myocardial necrosis in ICU patients with acute non-cardiac disease: a prospective study. Intensive Care Med.

[83] Ver Elst KM, Spapen HD, Nguyen DN, Garbar C, Huyghens LP, Gorus FK (2000). Cardiac troponins I and T are biological markers of left ventricular dysfunction. Clin Chem.

[84] Ammann P, Fehr T, Minder EI, Günter C, Bertel O (2001). Elevation of troponin I in sepsis and septic shock. Intensive Care Med.

[85] Ammann P, Maggiorini M, Bertel O (2003). Troponin as a risk factor for mortality in critically ill patients without coronary syndromes. J Am Coll Cardiol.

[86] Mehta NJ, Khan IA, Gupta V, Jani K, Gowda RM, Smith PR (2004). Cardiac troponin I predicts myocardial dysfunction and adverse outcome in septic shock. Int J Cardiol.

[87] Rudiger A, Grasser S, Fischler M, Hornemann T, Eckardstein A, Maggiorini M (2006). Comparable increase of b-type natriuretic peptide and amino-terminal pro-B-type natriuretic peptide in patients with severe sepsis, septic shock, and acute heart failure. Crit Care Med.

[88] Withaut R, Bush C, Fraunberger P (2003). Plasma atrial natriuretic peptide and brain natriuretic peptide are increased in septic shock: impact of interleukine-6 and sepsis associated ventricular dysfunction. Intensive Care Med.

[89] Charpentier J, Luyt C-E, Fulla Y (2004). Brain natriuretic peptide: a marker of myocardial dysfunction and prognosis during severe sepsis. Crit Care Med.

[90] Cuthbertson BH, Patel RR, Croal BL, Barclay J, Hillis GS (2005). B-type natriuretic peptide and the prediction of outcome in patients admitted to Intensive Care. Anaesthesia.

[91] Tung RH, Garcia C, Morss AM (2004). Utility of B-type natriuretic peptide for the evaluation of Intensive Care Unit shock. Crit Care Med.

[92] Forfia PF, Watkins SP, Rame E, Stewart KJ (2005). Relationship between B-type natriuretic peptide and pulmonary capillary wedge pressure in the Intensive Care Unit. J Am Coll Cardiol.

[93] Jefic D, Lee JW, Jefic D, Savoy-Moore RT, Rosman HS (2005). Utility of B-Type Natriuretic Peptide and N-terminal Pro B-Type Natriuretic Peptide in Evaluation of Respiratory Failure in Critically Ill Patients. Chest.

[94] Waijar S, Bonventre J (2007). Biomarkers for the diagnosis of acute kidney injury. Curr Opin Nephrol Hypertension.

[95] Hei ZQ, Li XY, Shen N, Pang HY, Zhou SL, Guan JQ (2008). Prognostic values of serum cystatin C and beta2 microglobulin, urinary beta2 microglobulin and N-acetyl-beta-D-glucosaminidase in early acute renal failure after liver transplantation. Chin Med J.

[96] Crenn P, Messing B, Cynober L (2008). Citrulline as a biomarker of intestinal failure due to enterocyte mass reduction. Clin Nutrition.

[97] Ruiz P, Tryphonopoulos P, Island E (2010). Citrulline evaluation in bowel transplantation. Transpl Proc.

[98] Piton G, Mamzon C, Monnet M (2010). Plasma citrulline kinetics and prognostic value in critically ill patients. Intensive Care Med.

[99] Lorente L, Martin MM, Labarta L (2009). Matrix metalloproteinase-9, -10, and tissue inhibitor of matrix metalloproteinases-1 blood levels as biomarkers of severity and mortality in sepsis. Crit Care.

[100] Yousef AA, Amr YM, Suliman GA (2010). The diagnostic value of serum leptin monitoring and its correlation with tumor necrosis factor-alpha in critically ill patients: a prospective observational study. Crit Care.

[101] Ware LB, Koyama T, Billheimer DD (2010). Prognostic and patho-genic value of combining clinical and biochemical indices in pa-tients with acute lung injury. Chest.

[102] Gattas DJ, Cook DJ (2003). Procalcitonin as a diagnostic test for sepsis: health technology assessment n the ICU. J Crit Care.

[103] Uzzan B, Cohen R, Nicolas P, Cucherat M, Perret GY (2006). Procalcitonin as a diagnostic test for sepsis in critically ill adults and after surgery or trauma: a systematic review and meta-analysis. Crit Care Med.

[104] Bouadma L, Luyt CE, Tubach F (2010). Use of procalcitonin to reduce patients´exposure to antibiotics in Intensive Care units (PROTATA trial): a multicentre randomised controlled trial. Lancet.

[105] Jung B, Embriaco N, Roux F (2010). Microbiological data, but not procalcitonin improve the accuracy of the clinical pulmonary infection score. Intensive Care Med.

[106] Durairaj L, Schmidt G (2008). Fluid therapy in resuscitated sepsis. Less is more. Chest.

[107] Payen D, de Pont A, Sakr Y (2008). A positive fluid balance is associ-ated with a worse outcome in patients with acute renal failure. Crit Care.

[108] Alsous F, Khamiees M, DeGirolamo A, Amoateng-Adjepong Y, Manthous CA (2000). Negative fluid balance predicts survival in patients with septic shock: a retrospective pilot study. Chest.

[109] Varon J, Fromm RE (2000). Fluid balance in sepsis. Are we ready for a negative balance?. Chest.

[110] Wenkui Y, Ning L, Jianfeng G (2010). Restricted peri-operative fluid administration adjusted by serum lactate level improved outcome after major elective surgery for gastrointestinal malignancy. Surgery.

[111] Winning J, Claus RA, Huse K, Bauer M (2006). Molecular biology on the ICU.From understanding to treating sepsis. Minerva Anestesiol.

[112] Claus RA, Otto GP, Deigner HP, Bauer M (2010). Approaching clinical reality: markers for monitoring systemic inflammation and sepsis. Curr Mol Med.

[113] Pene F, Courtine E, Cariou A, Mira JP (2009). Toward theragnostics. Crit Care Med.

[114] Iapichino G, Marzorati S, Umbrello M (2010). Daily monitoring of biomarkers of sepsis in complicated long-term ICU-patients: Can it support treatment decisions?. Minerva Anestesiol.

[115] Menitz PG, Reiter A, Jordan B, Lang T (2004). More interventions do not necessarily improve outcome in critically ill patients. Intensive Care Med.

